# The biosynthesis of pyoverdines

**DOI:** 10.15698/mic2018.10.649

**Published:** 2018-08-28

**Authors:** Michael T. Ringel, Thomas Brüser

**Affiliations:** 1 Institute of Microbiology, Leibniz University Hannover, Herrenhäuser Str. 2, 30419 Hannover, Germany.

**Keywords:** Pyoverdines, iron starvation, siderophores, Pseudomonas fluorescens, Pseudomonas aeruginosa, non-ribosomal peptide synthetases, periplasmic tailoring

## Abstract

Pyoverdines are fluorescent siderophores of pseudomonads that play important roles for growth under iron-limiting conditions. The production of pyoverdines by fluorescent pseudomonads permits their colonization of hosts ranging from humans to plants. Prominent examples include pathogenic or non-pathogenic species such as *Pseudomonas aeruginosa*, *P. putida*, *P. syringae*, or *P. fluorescens*. Many distinct pyoverdines have been identified, all of which have a dihydroxyquinoline fluorophore in common, derived from oxidative cyclizations of non-ribosomal peptides. These serve as precursor of pyoverdines and are commonly known as ferribactins. Ferribactins of distinct species or even strains often differ in their sequence, resulting in a large variety of pyoverdines. However, synthesis of all ferribactins begins with an L-Glu/D-Tyr/L-Dab sequence, and the fluorophore is generated from the D-Tyr/L-Dab residues. In addition, the initial L-Glu residue is modified to various acids and amides that are responsible for the range of distinguishable pyoverdines in individual strains. While ferribactin synthesis is a cytoplasmic process, the maturation to the fluorescent pyoverdine as well as the tailoring of the initial glutamate are exclusively periplasmic processes that have been a mystery until recently. Here we review the current knowledge of pyoverdine biosynthesis with a focus on the recent advancements regarding the periplasmic maturation and tailoring reactions.

## A BRIEF HISTORY OF PYOVERDINE RESEARCH

In 1892, Gessard discovered yellow-green fluorescent pigments of bacterial origin that he termed fluorescins 1. These pigments, which later have been renamed pyoverdines by Turfreijer 2, could be discolored by acidification, and they recovered their color upon neutralization 1. It was proposed by Turfitt in 1936 that the ability to produce these green pigments may be used for classification purposes 3. Pyoverdine-producing bacteria are known today to belong to the genus *Pseudomonas* and form a subgroup therein, referred to as “fluorescent pseudomonads”. Pyoverdines from various strains have also been termed “pseudobactins”, beginning with a publication that introduced this term based on claimed but not further detailed chemical and physical differences to known siderophores 4. Subsequent structural analyses did not reveal any distinguishing characteristics to pyoverdines 5. In fact, the “pseudobactin” structure was the first solved structure of a pyoverdine, but the name pseudobactin still occasionally occurs in literature.

**Figure 1:**
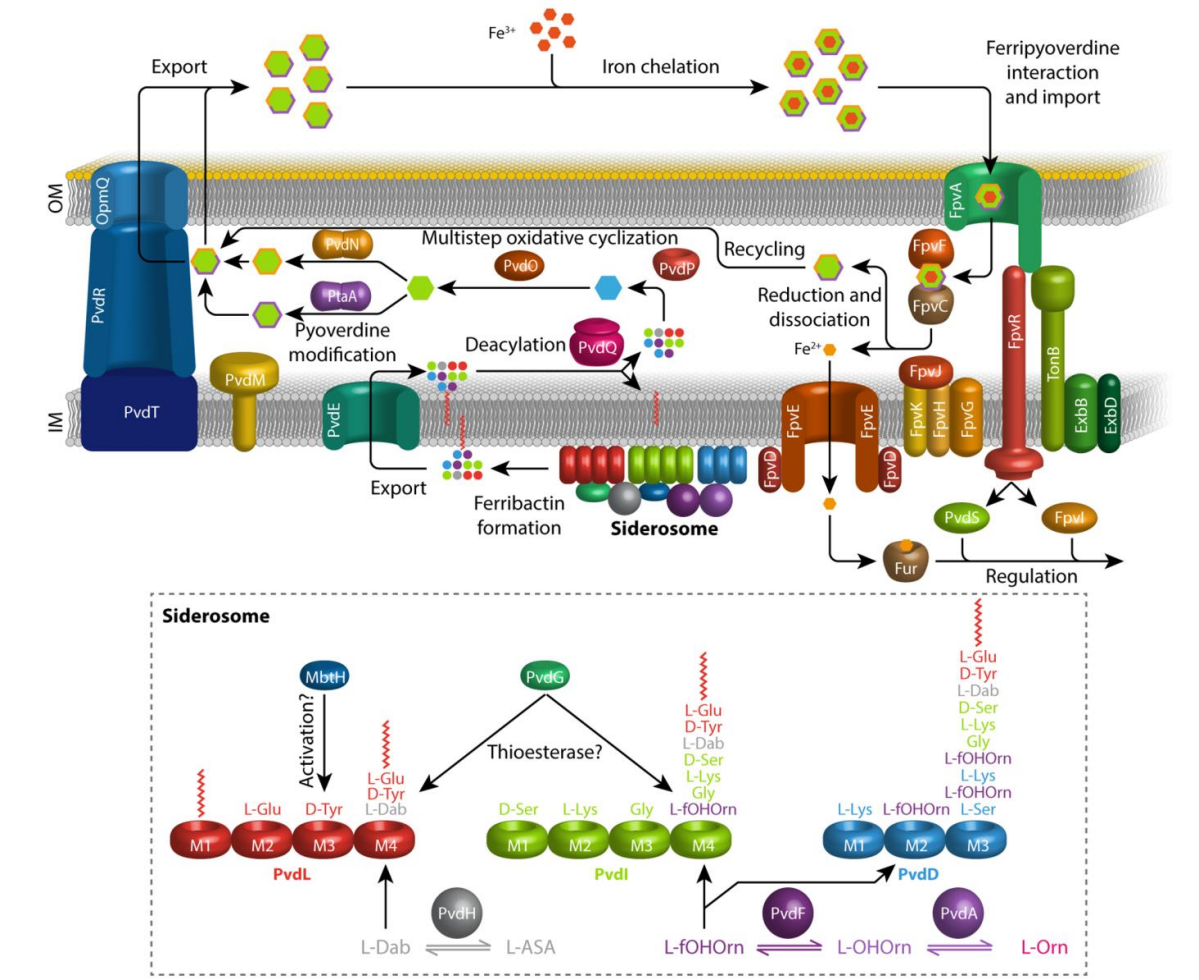
FIGURE 1: Current model for the biosynthesis, secretion, uptake and recycling of pyoverdines in *P. fluorescens *A506. The acylated ferribactin precursor is synthesized in the cytoplasm by NRPSs and auxiliary enzymes organized in membrane associated complexes termed “siderosomes”. The cytoplasmic synthesis is detailed in the box at the bottom. PvdL synthesizes the conserved N-terminal tripeptide with its acylation, the other NRPS are responsible for rest of the peptide and therefore vary between strains with distinct sequences. The auxiliary enzymes MbtH, PvdG, PvdH, PvdA, PvdF, and PvdD play the indicated roles (see text for details). The acylated ferribactin is exported most likely by PvdE into the periplasm, where it is deacylated by the Ntn-type hydrolase PvdQ. Subsequently, PvdP catalyzes the oxidative cyclization, resulting in dihydropyoverdine. PvdO, possibly in conjunction with other proteins, facilitates the final oxidation, yielding the characteristic pyoverdine chromophore. Thereafter, side-chain modification-pathways transform the original L-glutamic acid side chain either to the succinamide, catalyzed by PvdN, or the α-ketoglutarate, catalyzed by PtaA. The modified pyoverdines are then secreted via various transport systems such as PvdRT-OmpQ, and bind outside ferric iron. The complex binds to FpvA and is TonB-dependently taken up. FpvF and FpvC reduce and dechelate the iron, which is taken up by the FpvDE transporter. The apo pyoverdine is recycled. See text for details.

First functional insights were based on studies by King and coworkers who described in 1948 the induction of fluorescin (= pyoverdine) production by *P. aeruginosa* under iron limitation 6. Totter and Moseley found in 1952 that the production is inversely correlated with the logarithm of the iron concentration over a wide range 7. It took 26 years, until the next major progresses regarding pyoverdine function were made. In 1978, Meyer and coworkers first determined the extremely high affinity of pyoverdine to iron that is in the range of 10^32^ 8, before they finally revealed the function of pyoverdine in iron uptake 9. It was concluded that pyoverdines are siderophores, which are iron-binding chelators involved in iron-transport into the cell 10. In agreement with this function, it later turned out that the production of pyoverdines is tightly regulated in response to iron by the regulator Fur 11,12. This review focuses on the biosynthesis and secretion of pyoverdines as summarized in Figure 1. For a more detailed summary on the discovery and earlier studies of pyoverdines, the reader is referred to an earlier excellent review 13.

## CYTOPLASMIC INITIATION OF PYOVERDINE BIOGENESIS 

**Figure 2:**
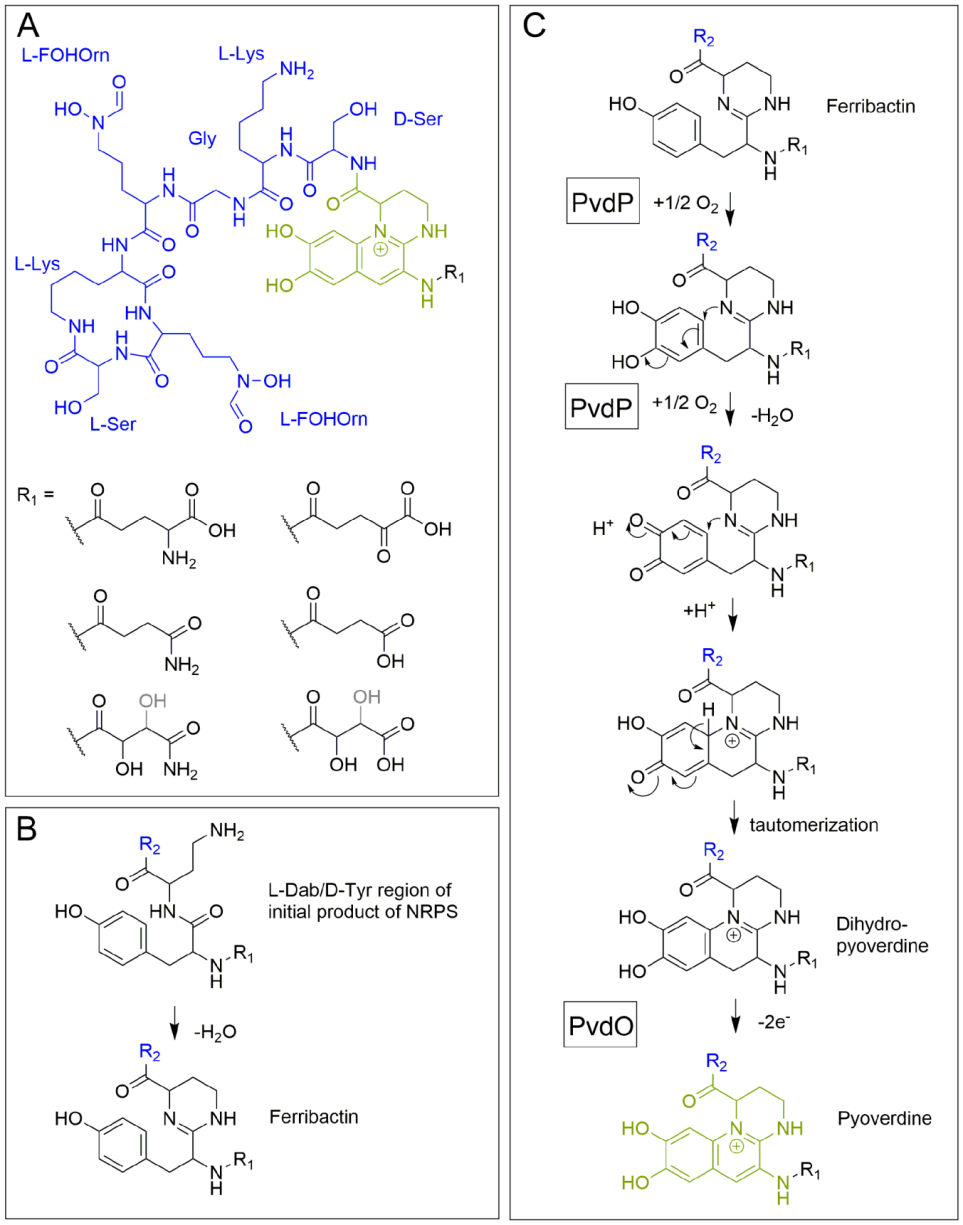
FIGURE 2: Structural aspects of pyoverdines. **(A)** The structure of pyoverdine from *P. fluorescens* A506. **(B)** The condensation occurring in ferribactins. **(C)** Current model of periplasmic pyoverdine fluorophore biosynthesis. Note that the exact position of the hydroxyl group (black/grey) in malamide or malic acid is not resolved. See text for details. R_2_ (blue) designates the peptide moiety.

The elucidation of the first pyoverdine structure 5 led to a new era in pyoverdine research that focused on the biosynthesis. Pyoverdines (Figure 2A) are generally composed of i) a characteristic 2,3-diamino-6,7-dihydroxyquinoline fluorophore, ii) a variable acyl side chain attached to the 3-amino group of the fluorophore, and iii) a strain-specific peptide backbone, usually bound to the C_1_-carboxyl group of the ring system (reviewed in 14,15), although so called isopyoverdines exist that have the peptide backbone attached to the C_3_-carboxyl group 16,17. The strain-specific peptide backbone varies in its sequence and can either be linear or (partially) intramolecularly cyclized. Furthermore, it can contain a number of unusual amino acids, such as β-hydroxy aspartic acid, β-hydroxy histidine, ornithine, cyclo-*N*_5_-hydroxy ornithine, *N*_5_-formyl-*N*_5_-hydroxy ornithine, *N*_5_-acetyl-*N*_5_-hydroxy ornithine and *N*_5_-hydroxybutyryl-*N*_5_-hydroxy ornithine, of which the hydroxamates or β-hydroxy carboxylates contribute to iron-chelation. Additionally, the amino acids in the peptide backbone may also be isomerized to the D-enantiomeric form 13,14.

Pyoverdine biogenesis starts in the cytoplasm, where non-ribosomal peptide synthetases (NRPSs) such as PvdL 18, PvdI 19 and PvdD 20,21 (depending on the strain) assemble an initially acylated ferribactin, the peptide precursor for pyoverdines 22. NRPSs are modular enzymes that add specific amino acids, one per module, to a growing peptide 23. Each module first adenylates its cognate amino acid at an adenylation domain and transfers it to a free thiol of a covalently bound phosphopantetheine cofactor. The peptide bonds are formed at condensation domains of the modules, thereby transferring growing peptides onto the phosphopantetheine-bound amino acid of the next module. Additional tayloring reactions and epimerizations can be catalyzed by specific domains 24. Finally, a thioesterase must cleave the thioester bond to release the peptide from the phosphopantetheine cofactor of the last module. Module 1 of PvdL incorporates either a myristic- or myristoleic acid side-chain instead of an amino acid as first building block 25,26, which is why ferribactin is acylated on the free amino group of the first incorporated amino acid. The first three amino acids of ferribactins, which are also incorporated by PvdL, are always L-glutamic acid (L-Glu), coupled via its γ-carboxy group to D-tyrosine (D-Tyr) and L-2,4-diaminobutyrate (L-Dab). This is important as the characteristic chromophore is derived from the D-Tyr and L-Dab residues, and we will later see that also the L-Glu residue at the N-terminus seems to be important, as several enzymes are produced that modify this residue.

The unusual amino acids in ferribactin are synthesized by pyoverdine-specific biosynthetic enzymes. L-Dab is produced by PvdH from L-aspartate β-semialdehyde (L-ASA) 27, whilst L-*N*_5_-formyl-*N*_5_-hydroxy ornithine (L-fOHOrn) is produced in two steps from L-ornithin by PvdA-dependent hydroxylation 28,29,30,31 and PvdF-dependent formylation 32. It has been proposed that all these enzymes, together with the NRPSs, may form a membrane associated complex termed “siderosome”, which could circumvent cytoplasmic toxicity 33,34.

Beside these enzymes, a small MbtH like protein has also been found to be associated with NRPSs of cytoplasmic ferribactin synthesis 35. The structure of this protein from *P. aeruginosa* has been solved and it was demonstrated to play a role in pyoverdine production or secretion 36. MbtH proteins have been shown to enhance the activity of NRPS adenylation domains to a variable extent 35,37, but the specific function of the MbtH like protein in ferribactin biosynthesis is unclear. Also, a soluble thioesterase PvdG has been implied to be involved in pyoverdine production 38. The corresponding gene *pvdG* is organized together with *pvdL*, and an interposon mutagenesis of *pvdG* abolished pyoverdine production 38. As only the last NRPSs of ferribactin synthesis, PvdD, has been shown to possess its own thioesterase active site motif 20, it may be that PvdG provides that functionality *in trans* for PvdL and possibly also for PvdI (Figure 1). PvdG might have overlapping function with a potential second soluble thioesterase (PA2411) 39, which could explain why its genetic inactivation did not abolish pyoverdine production 38. In specific strains, further auxiliary enzymes can be involved in cytoplasmic ferribactin biosynthesis steps. For example, there exist *P. aeruginosa* strains that produce a pyoverdine with an *N*-hydroxy-cyclo-ornithine residue, the so-called type-II pyoverdine, and a specific acylationprotein, PvdY_II_, is responsible for this 40. It is believed that most likely an acetylation of *N*-hydroxy-ornithine is required for the cyclization at some stage before the peptide is released from the NRPS 40.

## PERIPLASMIC COMPLETION OF PYOVERDINE SYNTHESIS

### Transport of the ferribactin precursor - some questions remain

The acylated ferribactin is most likely immediately translocated across the cytoplasmic membrane into the periplasm by the ABC transporter PvdE 41,42. The best evidence for this role comes from genetic studies that demonstrated abolished pyoverdine secretion in a PvdE interposon deletion mutant of strain *P. aeruginosa* PAO1, and the complementation of this phenotype by expression of the *pvdE* gene 42. Notably, that study showed that the *pvdE* mutant also did not display any periplasmic pyoverdine fluorescence anymore, indicating that PvdE must interfere with a step prior to fluorophore formation, which could well be ferribactin transport. A potential role in transport of the pyoverdine precursor has also been suggested in bioinformatic studies, that categorized PvdE to a class of ABC transporters for modified cyclic peptides 43. Interestingly, two older experimental studies had already inactivated the *pvdE* gene with somewhat different results. The inactivation of *pvdE* in *P. aeruginosa* OT11 resulted in complete absence of pyoverdine in culture supernatants 41, whereas a transposon mutagenesis study identified four clones of strain PAO1 MT1 with inactivating insertions in *pvdE* that all showed a specific lower fluorescence in the culture supernatant 44. However, this fluorescence was not acid-quenchable and thus the compound was not pyoverdine. Instead it was speculated to represent a precursor of pyoverdine or a degradation product 44. A transport of acylated ferribactin by PvdE has not yet been demonstrated *in vitro*, and the absence of fluorescence in the periplasm might certainly also have other reasons, albeit the transporter function is definitively the most likely scenario.

### Deacylation of the ferribactin precursor - quorum signaling meets pyoverdine production

In the periplasm, the acylation of ferribactin is removed by the Ntn-type hydrolase PvdQ 25,45. This now established function was first proposed by Visca *et al*. 23. PvdQ was initially discovered as quorum-quenching enzyme, which acts by deacylating the autoinducer *N*-(3-oxododecanoyl)-L-homoserine lactone (3-Oxo-C_12_-HSL) by hydrolysis of its amide bond 46. The enzyme is produced as a proenzyme that autoproteolytically cleaves itself in the periplasm by excising a 23-residue spacer forming an 18 kDa α-chain and a 60 kDa β-chain, which reassociate to form a functional heterodimer 46. It is an interesting aspect that pathways of quorum sensing and pyoverdine production converge in PvdQ, which is a true bifunctional enzyme. The function of PvdQ in quorum sensing of *P. aeruginosa* is believed to alter the ratio between the two autoinducers found in this species, namely the PvdQ substrate 3-Oxo-C_12_-HSL and the short-chain autoinducer *N*-butanoyl-L-homoserine lactone (C_4_-HSL), which is believed to specifically adapt gene expression to specific host environments 47. C_4_-HSL is not a substrate of PvdQ 48. A good example for effects of an altered ratio between the two autoinducers may be the effect of markedly reduced pyocyanine production in response to reduced 3-Oxo-C_12_-HSL levels 48. Pyocyanine generates oxidative stress and harms host organs 49. It thus might not be coincidental that pyoverdine production, which depends on the presence of PvdQ, is coupled with a reduction of host-threatening virulence: Both may serve to establish a permanent stable habitat in the host. A phenotypic analysis of a *pvdQ* deletion mutant of *P. aeruginosa* strain PAO1 indicated that PvdQ is required for swarming, biofilm formation and virulence 50. While the swarming and virulence phenotypes could be attributed to the absence of pyoverdine, the biofilm defect could not be suppressed by external addition of pyoverdine and thus may relate to quorum sensing. Interestingly, the production of PvdQ is tightly regulated and induced under iron-limiting conditions, which is another support for the idea that the effects of PvdQ on quorum sensing are specifically required when pyoverdines are produced 50. It would be good to know why all ferribactins initially need to be acylated and deacylated again after transport. One reason could be a membrane attachment that could help to anchor the precursor compound at the cytoplasmic membrane, which may improve the efficiency of the synthesis at clustered siderosomes or help to avoid its diffusion into the cytoplasm and thereby could guarantee its efficient transport. Another reason could be some mechanistic requirement for the transport system. A third reason may simply be the introduction of a biogenesis step that is controlled by an enzyme, which coordinates pyoverdine production with quorum sensing and virulence. As PvdQ influences the adaptation of fluorescent pseudomonads to host environments, it has been considered as target for inhibitors that hopefully may help to reduce the ability to thrive in hosts by blocking pyoverdine synthesis. After having solved the structure of PvdQ from *P. aeruginosa*, researchers succeeded in identifying such inhibitors, which were capable to inhibit growth of *P. aeruginosa* under iron-limiting conditions 51,52,53.

### Periplasmic formation of the fluorophore - the central roles of PvdP and PvdO

After ferribactin is deacylated, the fluorescent dihydroxyquinoline ring system is generated, which transforms the ferribactin into a pyoverdine. This ring system is strictly conserved in pyoverdines and provides a planar scaffold for two oxo-ligands of the siderophore. As mentioned before, ferribactin is always synthesized with the three N-terminal residues L-Glu/D-Tyr/L-Dab, and the tyrosine and diaminobutyrate residues of these form the fluorophore 54. In ferribactin, L-Dab 4-amino group is condensed with the carbonyl group of the neighboring D-Tyr (Figure 2B). An oxidative cyclization cascade as proposed by Dorrestein *et al*. 55 likely results in the formation of the three-ring fluorophore (Figure 2C). In that oxidative cyclization cascade, the tyrosine side chain is first hydroxylated to form a catechol that is then oxidized to an *o*-quinone. This facilitates the intramolecular addition, involving the 1-amino nitrogen atom from the neighboring L-Dab cycle and the quinone ring, resulting after tautomerization in the dihydro-dihydroxyquinoline system of dihydropyoverdine. The initial hydroxylation and oxidation steps are catalyzed by the copper-containing tyrosinase PvdP 56, which thereby also promotes the cyclization and formation of the dihydropyoverdine. This study observed a completion of the fluorescent fluorophore in the *in vitro* PvdP activity assays and suggested that PvdP is responsible for the complete fluorophore formation 56, which contrasted earlier studies that proposed the existence of an iron-enzyme-catalyzed oxidation of dihydropyoverdine 57. Also the detection of secreted dihydropyoverdine in a *Pseudomonas* strain supported the view that a single enzyme does not catalyze the complete conversion of ferribactin to the final fluorophore 58. Indeed, a rapid autoxidation can in principle complete the fluorophore under alkaline conditions 59, but recent studies demonstrated that this does not occur under weakly acidic physiological conditions, as found in the periplasmic space 60. It could be shown that a mutant strain lacking the putative oxidoreductase PvdO produced the dihydropyoverdines, indicating that PvdP catalyzes only the initial hydroxylation and first oxidation, whereas the final oxidation depends on PvdO 60. PvdO as purified from a heterologous *Escherichia coli* system did not contain a detectable cofactor and was inactive 60. It remains to be clarified whether active PvdO can be obtained that carries out the second oxidation alone, or whether it needs to associate with other components, such as specific electron transport systems or possibly the enzyme PvdP. Importantly, PvdO and PvdP always occur together, and currently both seem to be the only enzymes that are directly involved in the formation of the pyoverdine fluorophore 60.

As alternative to the above described oxidative cyclization cascade 55, a mechanism has been postulated based on the observation of a trihydroxylated “pseudoverdin” that was produced by a pyoverdine deficient mutant of *P. aeruginosa* PAO1, which was complemented by a cosmid gene bank clone to re-established fluorescence 61. The trihydroxylated “pseudoverdin” in that strain was not functional in iron acquisition and it was essentially an artificial system with unidentified genetic elements, but the resulting structure could permit an intramolecular Bucherer reaction as basis for the ring formation. Based on the identification of dihydropyoverdine-7-sulfonic acids, it was also proposed that a sulfonation can occur to facilitate this intramolecular Bucherer reaction 62,63. However, sulfonated dihydropyoverdines appear to be very lowly abundant and they might represent adducts of media constituents to the reactive dihydropyoverdine. As the conversion of dihydropyoverdine to pyoverdine most likely requires only one further enzyme, PvdO, and as the *pvdO* mutant did not accumulate a sulfonated dihydropyoverdine, we think that the Bucherer pathway is unlikely to be realized in this biosynthesis.

The electron transport pathways that are involved in the oxidation reactions are of fundamental importance and have not yet been clarified. Most relevant in this respect are studies with *P. fluorescens* ATCC17400, which show that the periplasmic membrane-associated oxidoreductase CcmC is involved 64,65,66. CcmC is well-known for its role as hemochaperone in periplasmic c-type cytochrome biogenesis 67,68,69,70, but it does not seem to be the cytochrome biogenesis role of CcmC that is relevant for its pyoverdine-related role. A very interesting mutagenesis study could dissect both functions of CcmC, indicating that distinct regions of this protein are involved in the two processes 66. The inactivation of CcmC has been described to reduce the level of thiol-oxidation in the periplasm, suggesting that oxidized thiols or more generally the oxidation-power in the periplasm may be relevant for pyoverdine formation 64. In this context, it is interesting that the addition of cysteine to the growing *ccmC* mutant culture resulted in formation of ferribactin 64. As it is now clear that PvdP is responsible for the conversion of ferribactin to dihydropyoverdine, it is likely that electrons must be transferred from PvdP to a periplasmic redox active compound that requires CcmC functionality.

### The side chain modifications - surprising periplasmicreactions catalyzed by PvdN and PtaA

**Figure 3:**
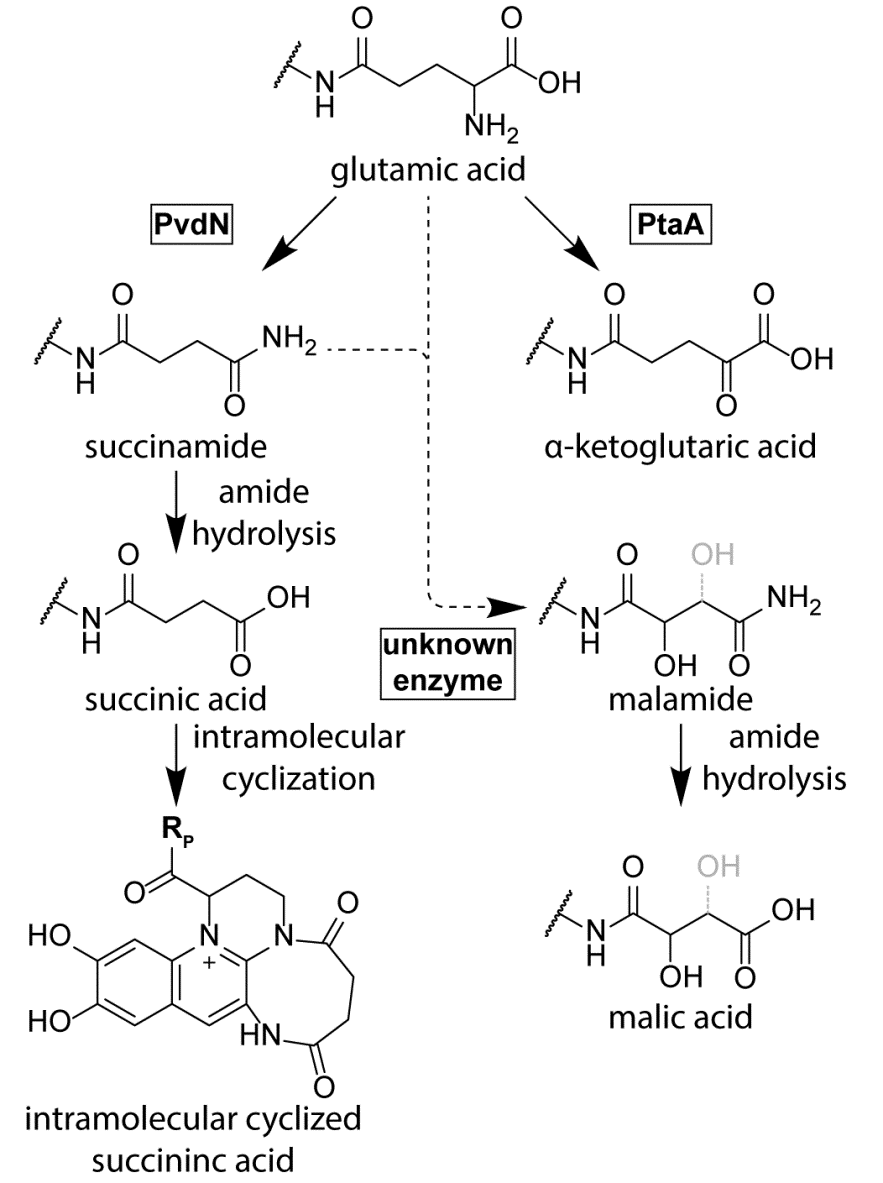
FIGURE 3: The branched periplasmic pathways for pyoverdine tailoring. PvdN generates the succinamide residue and PtaA the α-ketoglutarate residue from glutamate. An unknown enzyme converts succinamide (most likely) or possibly glutamic acid to malamide in some species. The lower abundant succinic acid and malic acid pyoverdine forms are likely hydrolysis products of the corresponding amides. Note that the exact position of the hydroxyl group in malamide and malic acid residues is not resolved.

While the conservation of the residues D-Tyr/L-Dab at positions 2 and 3 in all ferribactins is required for fluorophore biosynthesis, the conservation of the L-Glu at position 1 in ferribactins is not that easy rationalized. This acidic residue forms an amide bond via its γ-carboxylic group to D-Tyr and thus possesses free α-carboxy and α-amino groups. Importantly, mature pyoverdines usually do not contain these residues any more. Instead, depending on the strain analyzed, they usually possess a succinamide, succinate or α-ketoglutarate at this position, and sometimes malamide and malic acid are found, or even traces of intramolecular cyclized succinic acid (Figure 3). *P. aeruginosa*, for example, converts the glutamate completely to succinamide, succinate or α-ketoglutarate. It is unknown, why these modifications are made, but it has been speculated that they could play a role under specific environmental conditions or niches that could so far not be mimicked in pure cultures under laboratory conditions 71.

Until recently, no enzyme could be identified as being responsible for these “tailoring” modifications. The reason for this was the fact that the candidate proteins, the functionally uncharacterized periplasmic enzymes encoded in pyoverdine biosynthesis gene clusters, were believed to be essential for pyoverdine formation 38. Three of these periplasmic enzymes, PvdM, PvdN, and PvdO, are usually encoded in an operon. Interposon mutagenesis of the corresponding genes in *P. aeruginosa* indicated essential roles of these genes in pyoverdine biosynthesis, although it was already noted in the first of these studies that complementation analyses were required to exclude polar effects in the *pvdMNO* operon 38,42,72. In recent studies that used in frame deletions and complementations in *P. fluorescens* strain A506, which produces the same pyoverdine modifications as *P. aeruginosa*, it turned out that only the first gene in this operon is indeed essential, whereas PvdO is not essential, being responsible only for the final oxidation step of the fluorophore (see above), and the absence of PvdN resulted only in abolished formation of succinamide and succinic acid 71. This study therefore revealed the first tailoring enzyme for the periplasmic glutamic acid modification. PvdN is an interesting enzyme for two reasons: (i) It is a PLP-containing enzyme that catalyzes a new PLP-dependent reaction mechanism, a decarboxylation under retention of an amino group at the α-carbon atom 71, and (ii) it is transported by the twin-arginine translocation system as folded protein that requires cytoplasmic PLP binding to be transport competent 71,73. When the active site lysine is mutated, PvdN remains stuck in the membrane, most likely due to transport-incompatible, unfolded hydrophobic regions 71. As PvdN catalyzes the direct formation of succinamide from glutamate and is also required for the occurrence of succinate that is most likely the spontaneous hydrolysis product of succinamide, the formation of α-ketoglutarate from glutamate requires another enzyme, which recently turned out to be a novel PLP-containing transaminase, termed PtaA for “periplasmic transaminase A” 74. PtaA is usually encoded outside the large pyoverdine gene clusters, but an interesting exception has been recognized (*P. putida* H8234) where its gene substitutes the gene encoding PvdN. There are pyoverdine-producing pseudomonads that have both enzymes, PvdN and PtaA, some have only PvdN, and others only PtaA. Consequently, some strains produce the products of both biosynthesis branches, whereas others produce only products of one or the other branch 71,74.

PtaA is a “normal” PLP-containing transaminase. As PtaA is also present in a number of species incapable of pyoverdine production, it has been suggested to also be involved in other periplasmic biosynthesis pathways, which is why it received the general name “periplasmic transaminase A” 74. What is surprising and what makes this enzyme unusual is simply the fact that it is active in the periplasm. Like PvdN, PtaA is transported together with its PLP cofactor in a folded conformation via the twin-arginine protein translocation pathway 74. The cofactor thus enters the periplasm together with its protein in a tightly bound form, and it can therefore be assumed that the PLP is somehow regenerated from pyridoxamine after each reaction cycle inside the periplasm. Together, the recent advancements in the field established that there are two independent branches for the glutamate substitution in*P. aeruginosa* and *P. fluorescens* strains, which are (i) the PvdN-dependent branch that results in the succinamide and succinic acid as well as the intramolecular cyclization product, and (ii) the PtaA-dependent branch that results in α-ketoglutarate. There is nothing known about the biosynthesis of the rarely occurring malamide and malate forms of pyoverdines yet, which are not present in the current pyoverdine biosynthesis model organisms *P. aeruginosa* PAO1 and *P. fluorescens* A506. While it is likely that the malate is the hydrolysis product of the malamide, the malamide could in principle be formed by an unknown hydroxylating enzyme that acts on succinamide. The introduction of the hydroxyl group might also occur earlier, but a hydroxylated glutamate intermediate has never been found, suggesting some direct transformation of succinamide to malamide.

### Reactions of periplasmic pyoverdine biogenesis may not occur in a strict order

When PvdN is removed, PtaA converts all the pyoverdine to its α-ketoglutarate form. Conversely, the lack of PtaA results in the succinamide and succinic acid forms generated by PvdN. Removal of both tailoring enzymes leaves the original glutamate unaltered 74. It is tempting to assume that the tailoring steps occur after formation of the fully oxidized fluorescent pyoverdine. However, this may not be the case. The dihydropyoverdine formed by *pvdO* deletion strains is accepted by PvdN as well as by PtaA as the correspondingly modified dihydropyoverdine forms have been found 60. Apparently, the planarity of the attached ring system is not a prerequisite for substrate binding to these enzymes. Moreover, Budzikiewicz *et al*. detected a ferribactin with a succinamide side-chain 75, demonstrating that PvdN may even accept ferribactin as substrate, which shows that the ring system is not really relevant for the tailoring enzymes PvdN and PtaA. It is so far unknown to which extent the intermediates can be channeled from one enzyme to the next, but there is no evidence yet for such a channeling that could impose an order of events.

## GLOBAL TRADE ASPECTS - EXPORT, IMPORT, RECYCLING, AND REGULATION

### Export of pyoverdines - More than one way out 

After having summarized the knowledge about the enzymology of the biosynthesis steps, aspects of export, uptake, recycling and regulation need to be addressed to understand the physiology of pyoverdine biosynthesis. Pyoverdines can be exported via a transport system consisting of PvdR, PvdT, and OpmQ 76,77,78. This system has originally been shown to be involved in the secretion of pyoverdine that has been taken up from the environment and thus is recycled for multiple use 77,78, and later its involvement in transport of *de novo* synthesized pyoverdine was demonstrated 76. However, the PvdRT-OpmQ transporter cannot be the only export route, as strains mutated in this system are still able to secrete pyoverdines, albeit an accumulation in the periplasm has been demonstrated in such strains 76. Also a MexAB-OprM transporter has been implicated in pyoverdine secretion 79,80,81, but later studies indicate that the inactivation of this transporter had no significant effect on pyoverdine secretion 78. However, that study also indicated that the inactivation of the PvdRT-OpmQ system reduced pyoverdine secretion only to about 50-60%, which shows that also other systems must be involved. In *P. taiwanensis*, a type VI secretion system has been shown to mediate secretion of newly synthesized pyoverdine, and this study reports that the PvdRT-OmpQ system is not relevant for this process in their organism 82. Taken together, there is still considerable need to clarify under which conditions PvdRT-OmpQ systems are not only involved in recycling but also in secretion of *de novo* synthesized pyoverdines, and there is evidence that more not yet assigned transport systems are important.

### Import of pyoverdines - iron acquisition and siderophore recycling 

Organisms, especially those that live on or in host organisms, compete for the limited iron resources. Therefore, siderophore-uptake systems are usually specific for the siderophore that is used by the organism. Pyoverdines possess a highly variable, often even strain specific peptide moiety that confers distinguishable properties to the siderophore that can be taken up by differing uptake systems 13,83,84. Nonetheless, pyoverdine uptake systems exist that can use distinct pyoverdines of other strains 83. In the outer membrane, FpvA has been identified to be the ferripyoverdine receptor that recognizes iron-loaded pyoverdines 85,86. Its gene was cloned in 1993 87, and its structure was later elucidated in great detail 88,89,90. A number of mutational studies tried to elucidate the residues involved in pyoverdine binding, signaling and transport 91,92,93. In 2009 it could be demonstrated by Greenwald *et al*. 94, that the first amino acid residues in the peptide backbone of pyoverdine determine the binding affinity of pyoverdines to their cognate or non-cognate Fpv’s. The uptake is probably energized by the direct interaction of FpvA with the TonB-ExbBD complex at the energized inner membrane, which transduces sufficient energy to the outer membrane for transport 95,96. *P. aeruginosa* has two TonB homologs, and albeit TonB1 seems to be more important for iron uptake, TonB2 can partially fulfill the function of TonB1 97.

FpvA not only binds ferripyoverdine but also to the iron-free apo-pyoverdine, which is not imported but exchanged by ferripyoverdine that is then imported 98,99,100. The exchange appears to be accelerated by TonB 99. However, it has been suggested that the observation regarding binding of apo-pyoverdine by FpvA might be an artifact and that the detected binding could be due to trace-contaminations of aluminum chelates 101,102. Indeed, FpvA can bind a wide range of other pyoverdine-metal complexes that in case of Cu^2+^, Ga^3+^, Mn^2+^ and Ni^2+^ may even be imported, albeit with a reduced rate. Furthermore, pyoverdine chelates of Al^3+^, Cu^2+^, Ga^3+^, Mn^2+^, Ni^2+^ and Zn^2+^ can induce pyoverdine production by binding to FpvA 103. Some strains possess several FpvA homologs with distinct or overlapping specificities for pyoverdines. A very extensive investigation in this matter was performed by Hartney *et al*. in 2013, who demonstrated the specificity of a multitude of Fpv homologs and their pyoverdine-scavenging potential in *P. protegens* Pf-5 104.

After import, pyoverdine is not degraded or modified, nor is it imported into the cytoplasm. Instead, Fe^3+^ is reduced to Fe^2+^ periplasmically, liberated from pyoverdine, and taken up by the ABC transporter FpvDE 105. Involved components are encoded in the *fpvGHJK* and *fpvCDEF* operons 106. The inner membrane proteins FpvG and FpvH are essential for iron release, and there is indirect evidence that FpvG catalyzes the reduction step 106. The other components, such as FpvJ, FpvK, or even the ABC transporter FpvDE and its two soluble periplasmic binding proteins FpvC and FpvF, affect the release partially 106. As expected for a binding protein of a ferrous iron ABC transporter, there is indirect experimental evidence for chelation of ferrous iron by FpvC 106. The thus recycled apo-pyoverdine is reexported as described above into the extracellular compartment by OpmQ-PvdRT 77,78. It has also been reported that pyoverdine can be stored in the periplasmic compartment 42 but this process is not yet understood 107.

### Regulation of pyoverdine production - iron limitation and beyond 

**Figure 4:**
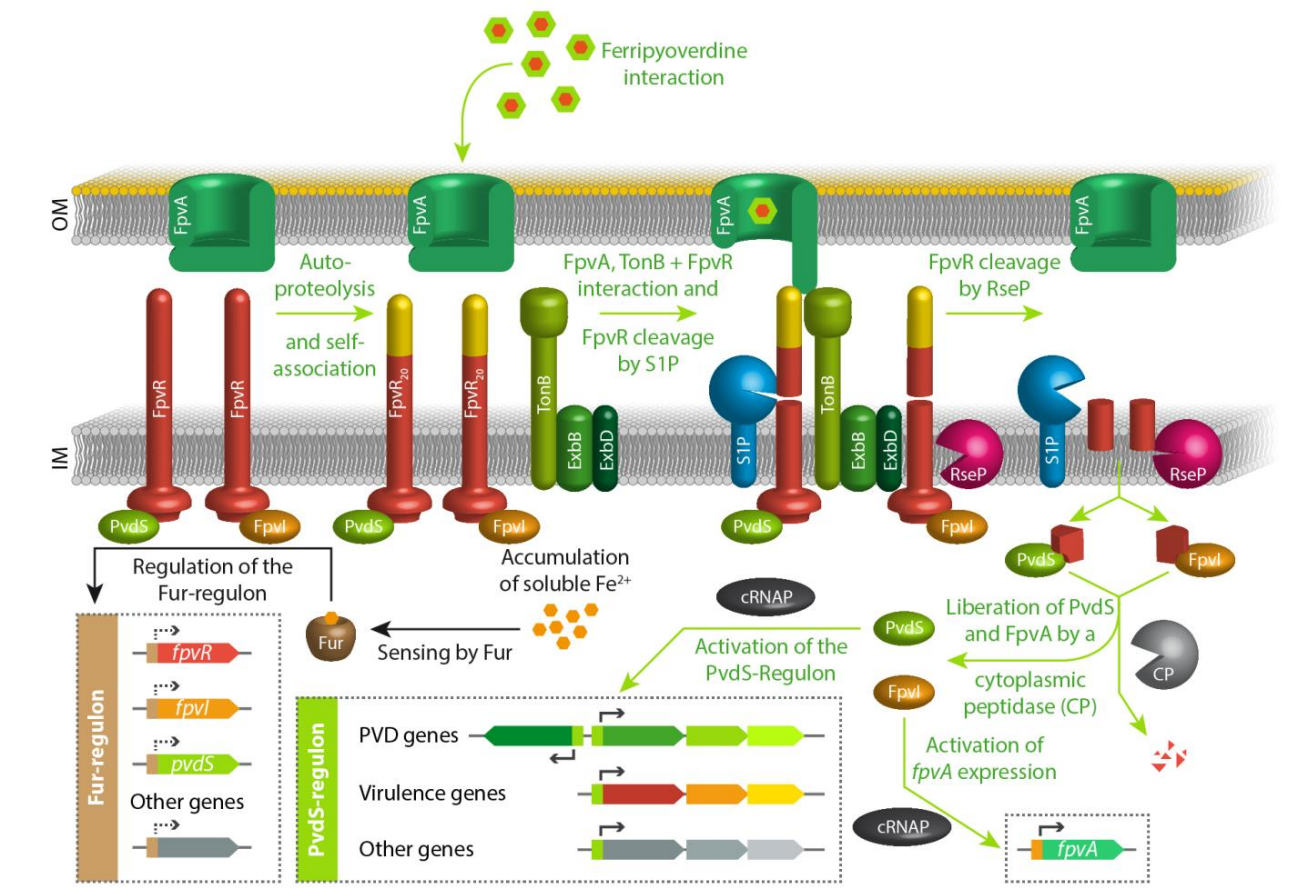
FIGURE 4: The key regulatory pathways for pyoverdine production in response to iron starvation. See text for details.

As mentioned in the introduction, iron starvation is the key signal for pyoverdine production. The regulator Fur senses ferrous iron ions in the cytoplasm and represses genes involved in iron uptake, including those encoding the regulatory proteins FpvR, FpvI, and PvdS 11,72,108,109,110. PvdS is a sigma factor required for the expression of pyoverdine biosynthesis genes and other, often virulence-related genes 111,112,113,114,115,116,117,118, FpvI is a sigma factor required for the genes encoding the outer membrane pyoverdine receptor/importer FpvA 119,120, and FpvR is an anti-sigma factor that binds to and thereby inactivates PvdS and FpvI 119,121. FpvR autoproteolytic cleaves itself at a periplasmic domain without any further degradation unless it contacts ferripyoverdine-bound FpvA 122,123. When this FpvR/FpvA contact occurs, which involves the activity of TonB (the transport-energizing inner membrane protein; see section on import above) 124, further proteolytic events that engage the protease RseP result in liberation of PvdS and FpvI and activation of their regulated genes 119,120,122,123,125,126. As the cascade begins with the sensing of a receptor-bound ferripyoverdine, it is noteworthy that some ferripyoverdine is required to activate the production of pyoverdine. In the absence of ferripyoverdine, the system therefore adjusts a basal level of PvdS and FpvI dependent gene expression, caused by a low abundance of FpvR, and this basal expression is required for the above described pyoverdine-dependent sensing pathway 127. In agreement with this view, mutants defective in pyoverdine production cannot upregulate the PvdS regulon under iron-limiting conditions 128,129. Finally, it is important to emphasize that the regulatory pathways for the production of pyoverdine play important roles beyond pyoverdine production. As mentioned above, the PvdS regulon also includes genes that are not involved in pyoverdine biosynthesis. In *P. aeruginosa*, such genes are clearly contributing to virulence, as the suppressed virulence of a *pvdA* deletion strain that lacks pyoverdines could be partially restored by deletion of the *fpvR* gene that encodes the anti-sigma factor FpvR 121. This *pvdA*/*fpvR* double mutant strain constitutively expresses the PvdS-dependent genes without producing pyoverdines. Pyoverdines that are initially sensed by the regulatory cascade thus can serve as signaling molecules in host environments. The above principal signaling pathway is summarized in Figure 4.

Interestingly, the regulation of pyoverdine biosynthesis is even more complex, because signals other than iron starvation have modulating effects. Among these are influences by the regulator CysB 130, which may imply a coordination with sulfur availability or biofilm formation and alginate production 131. Also phosphate starvation has been reported to trigger pyoverdine production in host environments 132,133. Additionally, the LexR type transcriptional regulator AmpR, which affects expression of more than 500 genes related to metabolism and virulence in *P. aeruginosa*, has recently been implicated in the regulation of pyoverdine production 134, and also the level of bis-(3’-5’)-cyclic dimeric guanosine monophosphate (c-di-GMP) is reported to modulate pyoverdine production 135.

### Concluding remarks

Pyoverdines play important roles for many pathogenic and non-pathogenic pseudomonads that thrive in host habitats. It is important to understand the biosynthesis of pyoverdines, and this review intends to give a brief survey about our current knowledge and the open questions. There are still some components unknown and some catalytic mechanisms not understood. Beside the basics of biosynthesis, the physiological aspects will become more important in future, as the process has to be understood in terms of cell biology, communication, and host interaction. One of these aspects is regulation, which seems to become more complex and interwoven with multiple other regulatory pathways, ranging from biofilm formation to nutrient supply. Future will reveal what else we can learn from pyoverdines.
